# Comparison of clinical outcomes of conjunctivo-mullerectomy for varying degrees of ptosis

**DOI:** 10.1038/s41598-023-46419-y

**Published:** 2023-11-05

**Authors:** Kanograt Pornpanich, Sunsri Shanokprasith, Pimkwan Jaru-ampornpan, Akarawit Eiamsamarng

**Affiliations:** Department of Ophthalmology, Faculty of Medicine Siriraj Hospital, Mahidol University, Siriraj, Bangkok Noi, Bangkok, 10700 Thailand

**Keywords:** Medical research, Outcomes research

## Abstract

To compare the success of conjunctivo-mullerectomy in patients with varying degrees of ptosis and identify factors affecting outcomes and complications. The prospective cohort was studied in patients with ptosis undergoing conjunctivo-mullerectomy with or without tarsectomy were enrolled. Ptosis was classified as mild, moderate, and severe per margin-to-reflex distance 1 (MRD1). Postoperative MRD1, complications, and 3-month success rates were evaluated. The study enrolled 258 ptotic eyes of 159 patients. Most eyes (233; 90.3%) achieved surgical success, 14 (5.4%) were overcorrected, and 11 (4.3%) were undercorrected. The success rates for mild, moderate, and severe ptosis were 96.6%, 91.7%, and 83.5%, respectively. The mild and moderate ptosis groups had a nonsignificant difference in success (− 4.9%; 95% CI − 12.0% to 4.5%; *P* = 0.36). However, the mild and severe ptosis groups’ rates significantly differed (− 13.1%; 95% CI − 23.6% to − 1.9%; *P* = 0.03). For all 3 ptosis groups, the success rates of individuals undergoing surgery without tarsectomy did not significantly differ. Patients undergoing conjunctivo-mullerectomy with tarsectomy had an increased risk of unsuccessful surgery (OR 3.103; 95% CI 1.205–7.986; *P* = 0.019). In conclusions, Conjunctivo-mullerectomy is safe and effective for all ptosis severities. The success rate was significantly lower for severe ptosis than mild or moderate ptosis. Levator muscle function was not associated with unsuccessful outcomes, but tarsectomy was.

## Introduction

Ptosis is an ophthalmic condition in which the upper eyelid margin is lower than usual. The upper eyelid normally covers 0.5 to 2 mm of the superior corneal limbus, with the highest curvature point nasal to the pupil center^1^. Ptosis can affect one or both eyes and is classified by etiology into 2 categories: congenital and acquired. Many causative mechanisms for ptosis have been described. They include an innervational defect (third cranial nerve palsy), Horner’s syndrome, underdevelopment or myopathy of the levator muscle, dehiscence or stretching of the levator aponeurosis, and the gravitational effect of mass at the upper eyelid. The ptotic condition affects patients’ appearances and visual function by limiting the superior visual field^2^. Three surgical procedures are typically used to correct ptosis: transcutaneous levator resection/advancement, conjunctivo-mullerectomy, and frontalis suspension^2^.

Muller’s muscle is responsible for upper eyelid elevation and is sympathetically innervated. Ptosis correction performed by conjunctivo-mullerectomy, which consists of the excision of Muller’s muscle and the overlying conjunctiva, has considerable advantages. They include ease of performance, the absence of unsightly scarring, a reduction in tissue trauma, precise and predictable postoperative outcomes, rapid recovery, and the preservation of the normal eyelid contour^3,4^. Theoretically, conjunctivo-mullerectomy is reserved for mild to moderate ptosis with good levator muscle function^5^. Nevertheless, the authors believe this surgical procedure could be used in cases of severe ptosis eyelids that are responsive to phenylephrine testing. A retrospective chart review by Patel et al.^6^ found that conjunctivo-mullerectomy with or without tarsectomy may be an alternative procedure for severe involutional ptosis correction. However, their study’s small sample size prevented success rates from being compared by the degree of ptosis.

The present investigation aimed (1) to compare the surgical success rate of conjunctivo-mullerectomy for varying degrees of ptosis and (2) to determine the factors affecting surgical outcomes and complications.

## Methods

Before this research began, the Siriraj Institutional Review Board, Mahidol University, Thailand, approved its protocol (COA no. Si 495/2016). All patients gave their written informed consent to participate, and the study fully complied with the Declaration of Helsinki. The prospective cohort study enrolled patients diagnosed with ptosis who were scheduled to undergo conjunctivo-mullerectomy with or without tarsectomy. The procedure was performed by oculoplastic specialists, oculoplastic fellowships, and residents in the Department of Ophthalmology, Siriraj Hospital, between April 2016 and February 2019.

Patients were enrolled if they were older than 15 years, were responsive to phenylephrine testing, had no history of previous eyelid or ptosis surgery, and could complete a 3-month follow-up. The exclusion criteria were patients with a phenylephrine allergy or a contraindication to a phenylephrine test (e.g., unstable cardiac disease or uncontrolled hypertension).

Jang et al.^7^ reported a surgical success rate of 83% for conjunctivo-mullerectomy. The sample size calculation was based on a 15% noninferiority margin and 95% CI (type I error 0.05; 1-sided). The population was determined to be 83 eyes per group (mild, moderate, and severe cases of ptosis).

Data were collected on patients’ demographic profiles, ptosis etiologies, preoperative margin-to-reflex distance 1 (MRD1), amount of MRD1 change in phenylephrine testing, levator muscle function, and presence of preoperative lagophthalmos.

The preoperative MRD1 values were obtained by directing light from 1 m in front of the patients’ corneas. The measurements were made in millimeters from the upper lid margin to the corneal light reflex. The patients were seated, and their eyes were in the primary gaze. The patients were classified into 3 groups according to their preoperative MRD1: mild (MRD1 2–3.5 mm), moderate (MRD1 0.5–1.5 mm), and severe (MRD1 ≤ 0 mm) ptosis.

In the phenylephrine test, 1 drop of 0.5% tetracaine was instilled into each tested eye. The examiner’s finger elevated the upper eyelid with the eye in a downgaze position, and then 1 drop of 10% phenylephrine was instilled at the superior limbus of the eye. MRD1 was assessed before and 10 min after phenylephrine instillation. The Phenylephrine test was done for each eye separately in bilateral cases to neutralize the Hering law effect. A “positive response” to the phenylephrine test was defined as an MRD1 value that was more than 0.5 mm above the pre-instillation value.

The “predicted MRD1” was the anticipated MRD1 after the conjunctivo-mullerectomy procedure. In this study, the procedure was performed using the technique proposed by Perry et al.^8^ The amount of resection was determined by the following formula:

$$ {{9 mm of conjunctiva and Muller}}^{\prime}{\text{s muscle }} + {{ x mm of tarsectomy}}, $$

where “x” is the distance of the undercorrection after phenylephrine testing. The formula is based on the hypothesis that 10% phenylephrine instilled into a tested eye maximally stimulates its Muller’s muscle. Excision of 9 mm of conjunctiva and Muller’s muscle is nearly a complete excision and should elevate the eyelid to the same level as for the phenylephrine test. If the MRD1 value after the phenylephrine test was still less than for the other eye, the tarsus was resected by the amount of undercorrection. To preserve tarsal stability, tarsectomies over 2.5 mm in length were not performed.

During the surgical procedure^6,9^, 0.5% tetracaine was instilled into the operated eye, and 2% lidocaine with 1:100,000 epinephrine was injected into the upper palpebral conjunctiva. A 4-0 silk traction suture was placed in the middle of the upper lid 2 mm from its margin, with the suture passing through the tarsus and skin. The upper lid was then everted over a Desmarres lid retractor to expose the palpebral conjunctiva. A caliper was used to measure a distance of 4.5 mm from the superior tarsal border to the superior fornix. Toothed locking forceps were used to grasp the palpebral conjunctiva and Muller’s muscle and separate them from the underlying levator aponeurosis. A Putterman Muller’s muscle-conjunctival resection clamp was placed at the superior tarsal border. If a tarsectomy was needed, the desired amount of tarsus was incorporated into the clamp. A double-armed 6-0 Prolene suture was passed in a running horizontal mattress suture technique 1.5 mm below the clamp from the temporal to the nasal side. Both arms of the suture were passed through the skin. Muller’s muscle and the palpebral conjunctiva were cut with a number 15 surgical blade between the running suture and the clamp. The running horizontal mattress suture was tightened to reapproximate the conjunctival edge. Both arms of the suture were tied externally. Concurrent blepharoplasty was performed in some patients with significant dermatochalasis, depending on the surgeon’s preference. Postoperatively, patients received topical antibiotics 4 times per day for 1 week.

Follow-ups were carried out after 1 week, 1 month, and 3 months. The postoperative MRD1 values and any complications (such as lagophthalmos and ocular staining) were recorded at each visit. The surgical success rates of the 3 groups (mild, moderate, and severe cases of ptosis) were compared. “Surgical success” was defined as a difference of 1 mm or less between the predicted MRD1 value and the postoperative MRD1 value at the 3-month visit. “Overcorrection” or “undercorrection” was defined as differences of more than 1 mm between the predicted and the postoperative MRD1 values, respectively. A subgroup analysis examined factors potentially affecting surgical outcomes. The factors were age, sex, cause of ptosis, preoperative MRD1, ptosis severity, levator function, concurrent tarsectomy, blepharoplasty procedure, and postoperative complications.

### Statistical analysis

PASW Statistics for Windows, version 16, was used for all analyses. Values are presented as the mean ± SD (range) for continuous variables or as the number (percentage) of subjects for categorical variables. The surgical success rates of the 3 groups of patients were compared and are presented as differences with 95% confidence intervals. The preoperative factors associated with the surgical results were compared using a chi-squared test for categorical data and an unpaired t-test or Mann–Whitney U test for continuous data. Probability (*P*) values < 0.05 indicated statistical significance.

## Results

A total of 258 eyes of 159 patients with ptosis were recruited. The demographic data of the patient population are listed in Table 1. The average age was 58 years (range 15 to 88), and female patients (129; 81.1%) outnumbered male patients (30; 18.9%). The ptosis was mostly acquired in etiology (96.2%). Sixty patients (37.74%) were identified with unilateral ptosis, while 99 patients (62.26%) had bilateral ptosis.

**Table 1:** Demographic data of patients.

Number of patients	159
Number of eyes	258
Sex	
Male	30 (18.9%)
Female	129 (81.1%)
Age	
Mean (± SD), years	58.16 (± 17.06)
Range	15–88
Cause	
Congenital	6 (3.8%)
Acquired	153 (96.2%)
Type	
Unilateral	60 (37.74%)
Bilateral	99 (62.26%)

Patients were categorized into 3 groups, depending on their preoperative MRD1 values. There were 58 eyes (22.5%) with mild ptosis, 121 eyes (46.9%) with moderate ptosis, and 79 eyes (30.6%) with severe ptosis. A comparison of the preoperative parameters by the degree of ptosis is presented in Table 2. There were statistically significant differences in sex, mean age, and preoperative MRD1. The mean preoperative MRD1 values for the mild, moderate, and severe ptosis groups were 2.23 ± 0.46 mm (range 2.0 to 4.0), 1.15 ± 0.38 mm (range 0.5 to 1.5), and − 0.68 ± 0.99 mm (range − 4.0 to 0), respectively (*P* < 0.001). The incidence of preoperative lagophthalmos was 2 eyes, with 1 eye from the mild group and the other from the severe group.

**Table 2:** Comparison of preoperative parameters by the degree of ptosis.

	Mild	Moderate	Severe	Total	*P* value
Number of eyes n (%)	58 (22.5%)	121 (46.9%)	79 (30.6%)	258 (100%)	
Sex					
Female n (%)	52 (89.7%)	115 (95%)	60(75.9%)	227 (88%)	** < 0.001**
Cause					
Congenital n (%)	2 (3.4%)	1 (0.8%)	3 (3.8%)	6 (2.3%)	0.360
Acquired n (%)	56 (96.6%)	120 (99.2%)	76 (96.2%)	252 (97.7%)	
Age					
Mean ± SD, years	53.5 ± 16.0	58.17 ± 15.24	64.47 ± 15.41	59.05 ± 15.93	** < 0.001**
Range, years	7–80	7–82	8–88	7–88	
Preoperative MRD1					
Mean (± SD), mm	2.23 ± 0.46	1.15 ± 0.38	− 0.68 ± 0.99	0.83 ± 1.26	** < 0.001**
Range	2.0 to 4.0	0.5 to 1.5	− 4.0 to 0	− 4.0 to 4.0	
Preoperative lagophthalmos n (%)	1 (1.7%)	0	1 (1.3%)	2 (0.8%)	0.500

Statistically significant P values are shown in bold.

The overall surgical success rate of conjunctivo-mullerectomy was 90.3% (233 eyes). The mean change in the MRD1 value was 2.38 ± 1.21 mm (range 0 to 6.5). The highest success rate was 96.6% (56 eyes) for the mild degree of ptosis, followed by 91.7% (111 eyes) and 83.5% (66 eyes) for the moderate and severe degrees, respectively. There was no significant difference in the success rates of the mild and moderate ptosis groups (difference − 4.9%; 95% CI − 12.0 to 4.5%; *P* = 0.36). In contrast, there was a significant difference between the success rates of the mild and severe ptosis groups (difference − 13.1%; 95% CI − 23.6 to − 1.9%; *P* = 0.03). Of the 25 eyes that failed to achieve surgical success, 14 (5.4%) were overcorrected, and 11 (4.3%) were undercorrected.

A comparison of the parameters of the surgical-success and surgical-failure groups is shown in Table 3. The preoperative mean MRD1 of the surgical-success group (0.903 mm ± 1.20; range − 4.0 to 4.0) was significantly higher than that of the surgical-failure group (0.16 mm ± 1.64; range − 4.0 to 3.0; *P* = 0.006). Seventy-one of the 233 eyes (30.5%) in the surgical-success group and 16 of the 25 eyes (64%) in the surgical-failure group received conjunctivo-mullerectomy with concurrent tarsectomy. A significantly higher proportion of eyes underwent concurrent tarsectomy in the surgical-failure group (*P* = 0.001). Nevertheless, there was a nonsignificant difference in the tarsectomy length of the 2 groups (surgical-success group, 1.25 mm ± 0.69; surgical-failure group, 1.28 mm ± 0.45; *P* = 0.913). Similarly, the 2 groups had no significant difference in their levator function (*P* = 0.735). The proportion of patients in the groups undergoing concurrent blepharoplasty also did not differ (surgical-success group, 48.1%; surgical-failure group, 60%; *P* = 0.260).

**Table 3:** Comparison of the parameters of the surgical-success group and the surgical-failure group.

	Surgical success group	Surgical failure group	*P* value
Number of eyes	233	25	
Sex			
Female (n, %)	205 (88%)	22 (88%)	0.998
Cause			
Acquired (n, %)	228 (97.9%)	24 (96%)	0.565
Age			
Mean ± SD, years	58.70 ± 16.12	62.32 ± 13.88	0.257
Preoperative MRD1			
Mean (± SD), mm	0.903 (± 1.20)	0.16 (± 1.64)	**0.006**
Range	− 4.0 to 4.0	− 4.0 to 3.0	
Degree of ptosis			
Mild	56 (24.0%)	2 (8.0%)	**0.044**
Moderate	111 (47.6%)	10 (40.0%)	
Severe	66 (28.3%)	13 (52.0%)	
Tarsectomy			
Yes	71 (30.5%)	16 (64.0%)	**0.001**
No	162 (69.5%)	9 (36.0%)	
Length of tarsectomy			
Mean (± SD), mm	1.25 (± 0.69)	1.28 (± 0.45)	0.913
Levator function (n, %)			
Poor (≤ 4 mm)	4 (1.7%)	1 (4.0%)	0.735
Fair (5–7 mm)	10 (4.4%)	2 (8.0%)	
Good (8–12 mm)	145 (62.0%)	15 (60.0%)	
Excellent (≥ 13 mm)	74 (31.9%)	7 (28%)	
Blepharoplasty			
Yes	112 (48.1%)	15 (60.0%)	0.260
No	121 (51.9%)	10 (40.0%)	

Statistically significant P values are shown in bold.

A multiple logistic regression analysis showed that conjunctivo-mullerectomy combined with tarsectomy was associated with an increased risk of unsuccessful surgery (OR 3.103; 95% CI 1.205 to 7.986; *P* = 0.019). However, there was no significant association between the degree of ptosis and the surgical outcomes (Table 4).

**Table 4:** Independent factors affecting successful surgical outcomes.

	Adjusted OR	Surgical-failure group	*P* value
Tarsectomy	3.103	1.205–7.986	**0.019**
Degree of ptosis (compared with mild ptosis)			
Moderate	1.579	0.592–4.213	0.362
Severe	3.199	0.654–15.643	0.151

Statistically significant P values are shown in bold.

A subgroup analysis of patients who underwent surgery without tarsectomy revealed that the overall surgical success rate was 94.7% (162 eyes). The highest success rate was 96.6% for a moderate degree of ptosis, followed by 96.1% and 87.9% for mild and severe degrees, respectively. There were no significant differences in the success rates of these 3 groups (Table 5).

**Table 5:** Comparison of surgical outcomes by the degree of ptosis (without tarsectomy).

	Mild	Moderate	Severe	Total
Number of eyes	51	87	33	171
Success rate (n, %)	49 (96.1%)	84 (96.6%)	29 (87.9%)	162 (94.7%)

Of the eyes that failed to achieve surgical success, 1 of the 14 overcorrected eyes and 4 of the 11 undercorrected eyes needed a second operation. The remaining eyes in the overcorrected and undercorrected groups were cosmetically acceptable.

The conjunctivo-mullerectomy complications were lagophthalmos (16 eyes; 6.2%) and punctate epithelial erosion (40 eyes; 15.5%). None needed treatment.

## Discussion

Many surgical techniques have been described for ptosis correction. The choice of technique is related to the severity of ptosis, the levator muscle function, and the response to the phenylephrine test.

Transcutaneous levator resection/advancement is an effective procedure for correcting ptosis, with the literature reporting success rates of 70% to 95%^10–12^. The advantages of the procedure are its ability to correct moderate to severe degrees of ptosis with fair levator function and to adjust lid height intraoperatively. However, many studies found that conjunctivo-mullerectomy produced better cosmetic outcomes with fewer lid contour abnormalities and a more predictable postoperative eyelid height than the levator advancement procedure^3,4,13^. Although conjunctivo-mullerectomy is typically reserved for treating mild to moderate ptosis with good levator function, some studies have found that the procedure may also be an option for severe involutional ptosis correction^6^.

**Figure 1:**
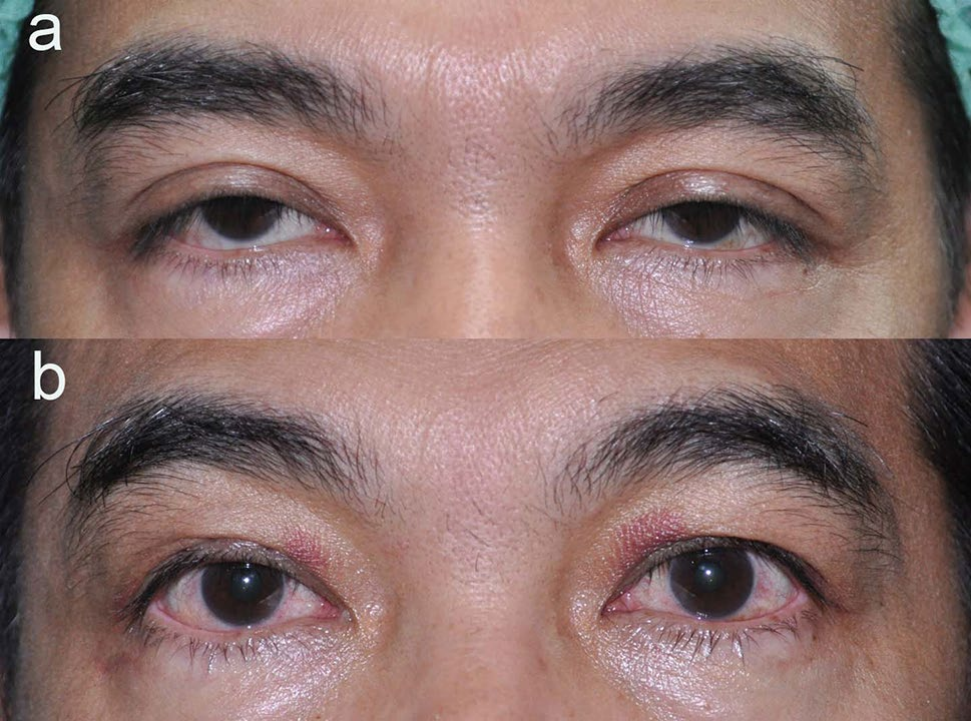
Representative photographs of a 47-year-old male with bilateral severe ptosis who underwent conjuctivo-mullerectomy without tarsectomy on both eyes. (**a**) Pre-operative MRD1 was − 0.5 mm in the right eye and − 1.0 mm in the left eye, (**b**) at 1-week post-operative follow-up.

Our study found that the overall surgical success rate of conjunctivo-mullerectomy was 90.3%, which is noticeably higher than the 83% and 87% of 2 earlier studies^7,8^. Our mean MRD1 change was 2.38 ± 1.21 mm (range 0 to 6.5). A recent study on involutional ptosis reported success rates of 88% for mild to moderate ptosis and 70.2% for severe ptosis, but there was no significant difference (*P* = 0.03)^14^. These findings contrast with those of Sweeney et al.^15^, who found a higher success rate for severe ptosis (97.2%) than for mild to moderate ptosis (90.9%; *P* = 0.42). In our study, the highest success rate was 96.6% for the mild degree of ptosis, followed by 91.7% and 83.5% for the moderate and severe degrees, respectively. Additionally, there was a statistically significant difference between the success rates of our mild and severe ptosis groups. Figure 1 demonstrates a favorable surgical outcome in a severe ptosis patient in this study. Nevertheless, the varying definitions of surgical success used by the published studies may have affected their apparent success rates.

In our study, we found that the mean change in MRD1 for severe ptosis was 3.25 ± 1.17 mm (range 0 to 6.5), which was close to the value of 3.65 mm (range 0.5 to 7.5) reported by Patel et al.^6^ All subjects in our study were Asian, whereas almost all previous studies were Caucasian. The eyelid and tarsal anatomies of Asians and Caucasians differ, with suborbicular and preaponeurotic fat typically more abundant in Asian eyelids^16^. These differences may affect anticipated surgical outcomes. We suggest that a larger number of severe ptosis cases should be studied to determine the correlations between the amount of conjunctiva, Muller’s muscle, tarsal resection, and the amount of eyelid elevation in the Asian population.

Some previous studies^7,17^ showed more undercorrection than overcorrection outcomes after conjunctivo-mullerectomy. Other investigations found only undercorrection^8,13^. By contrast, we found overcorrection (14 eyes; 5.8%) and undercorrection (11 eyes; 3.9%), with a higher incidence of overcorrection. However, the overall reoperation rate in our study was only 1.9% (5 of 258 eyes; 0.3% for overcorrection and 1.6% for undercorrection). Technically, another 20 eyes (13 overcorrected and 7 undercorrected) failed to meet our study’s definition of surgical success. Despite that, the eyes were considered cosmetically acceptable to the patients and the clinicians.

**Figure 2:**
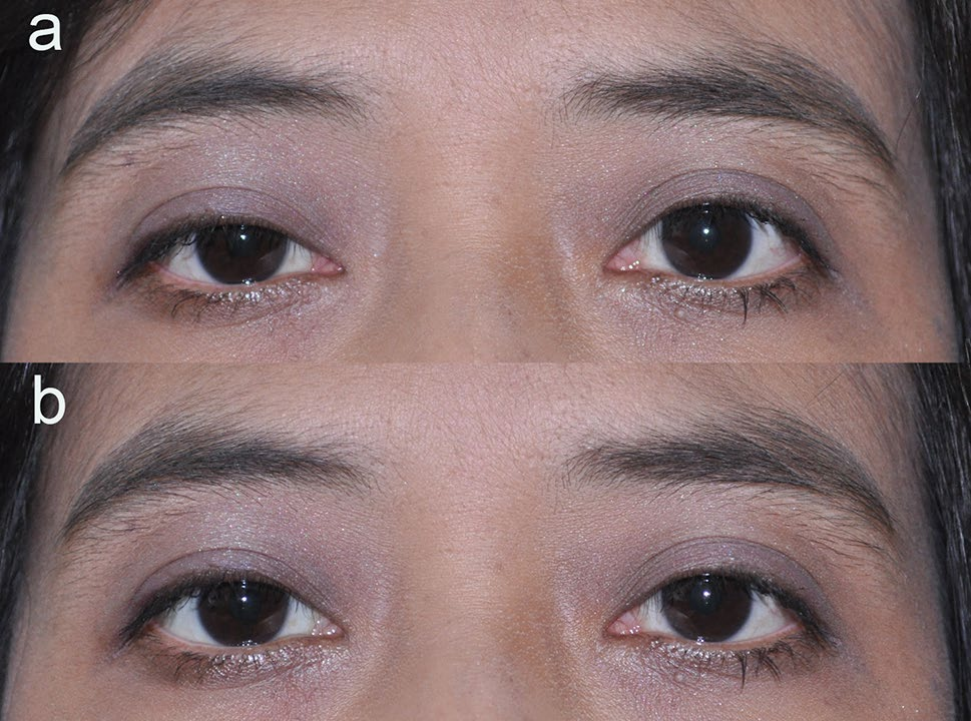
Representative photographs of a 38-year-old female with unilateral moderate congenital ptosis and fair levator function on the right eye underwent conjuctivo-mullerectomy without tarsectomy. (**a**) The pre-operative MRD1 was 1.5 mm and levator function was 6 mm in the right eye. (**b**) At 1-week post-operative follow-up.

Significant (*P* < 0.05) factors associated with successful surgery have previously been proposed to be female sex, concurrent lower blepharoplasty, and higher preoperative MRD1^18^. The current investigation found that concurrent tarsectomy was associated with an increased risk of unsuccessful surgery (OR 3.103; 95% CI 1.205 to 7.986; *P* = 0.019). However, the association did not depend on the length of the tarsectomy. Our study also found no correlation between levator function and surgical outcome. Two previous reports^19,20^ showed favorable outcome from conjunctivo-mullerectomy in poor to fair levator function patients, which was supported by some of our patients who had fair levator function (Fig. 2). Therefore, the surgical success of conjunctivo-mullerectomy may not depend on good levator function in case of positive phenylephrine test. However, earlier studies^3,21–23^ excluded poor levator function patients from their investigations. Hence, the effectiveness of conjunctivo-mullerectomy in poor levator function needs to be further studied.

One issue with conjunctivo-mullerectomy is that its posterior approach may worsen dry eyes by damaging the otherwise healthy conjunctiva or accessory lacrimal gland^24,25^. Evidence supporting this concern has been provided by Beaulieu et al.^26^, who reported that there was goblet cell depletion in the conjunctiva overlying the region of surgery. However, Bruna et al.^27^ found that combining conjunctivo-mullerectomy with upper eyelid blepharoplasty did not worsen ocular surface scores or dry eye symptoms. Additionally, Dailey et al.^28^ determined that conjunctivo-mullerectomy did not affect tear production. Another study ascertained no changes in the ocular surface disease parameters of tear break-up time, lipid layer thickness, and osmolarity after this surgery^29^. Our results agree with these prior findings in that we found punctate epithelial erosion in 40 eyes (15.5%) without significant symptoms or the need for treatment. Although postoperative lagophthalmos was present in 16 eyes (6.2%; range 0.5 to 3 mm), there were no serious adverse effects on the ocular surface or visual outcomes.

Our research has several limitations. One is its variation in MRD1 evaluations. Because many clinicians obtained them, there may have been measurement bias. Furthermore, varying levels of surgical experience may have affected the surgical outcomes. Moreover, all patients are Asian, so our finding may not represent for all ethnicities. Another limitation is the short follow-up period of 3 months. The authors intend to measure the MRD1 by photograph, to draw upon a larger population and a single surgeon, and to extend the follow-up time in a future study.

In summary, the success rate of conjunctivo-mullerectomy in cases of severe ptosis was significantly lower than that in cases of mild and moderate ptosis. Nevertheless, it was still reasonably high, at 83.5%. For all degrees of ptosis, conjunctivo-mullerectomy presented no serious complications, and the reoperation rates were low. Consequently, conjunctivo-mullerectomy can be considered an alternative procedure for all degrees of severe ptosis without concern for levator function.

## Data Availability

The datasets generated and analyzed during the study are available from the corresponding author upon reasonable request.
